# Janus or homogeneous nanoparticle mediated self-assembly of polymer electrolyte fuel cell membranes

**DOI:** 10.1039/c8ra03187h

**Published:** 2018-05-22

**Authors:** Yusei Kobayashi, Noriyoshi Arai

**Affiliations:** Kindai University 3-4-1 Kowakae Higashiosaka Osaka Japan kobayashiyusei@gmail.com +81 6 4307 3483 +81 6 6727 2024

## Abstract

The functionality of polymer electrolyte fuel cell membranes depends on the self-assembled structure of the graft polymer. To control self-assembly, nanoparticles (NPs) are often used as catalysts. Hence, we investigate the effect of hydrophilic (HI), hydrophobic (HO), and Janus nanoparticles (JNPs) for the self-assembly of graft polymers using dissipative particle dynamics (DPD) simulations. We found that the differences that appeared among the self-assembled structures of water depended on the concentration of PEFC. We also calculated the diffusion constant of water (D(H_2_O)) from the slopes of the time-averaged mean square displacement (MSD) curves. HI NPs had the largest effect in suppressing the diffusion of water because the HI NPs incorporated into the water particles. It was also seen that D(H_2_O) with various NPs gradually decreased as the number of NPs increased for three PEFC concentrations (70%, 80%, and 90%). Thus, a close correlation between the position and chemical composition of NPs in polymer electrolyte fuel cell (PEFC) membrane systems has been found. Moreover, the mean square radius of gyration 〈*R*_g_〉 and the mean square end-to-end distance 〈*R*〉 was calculated to analyse the self-assembled structures of PEFC. The 〈*R*_g_〉 and 〈*R*〉 increased as the concentration of PEFC was increased, with and without various NPs.

## Abstract 2

We investigated the effect of the chemical nature of nanoparticles for the self-assembly of graft polymers. Hydrophilic nanoparticles had the largest effect in suppressing the diffusion of water because it is incorporated into the water particles.

## Graphical abstract

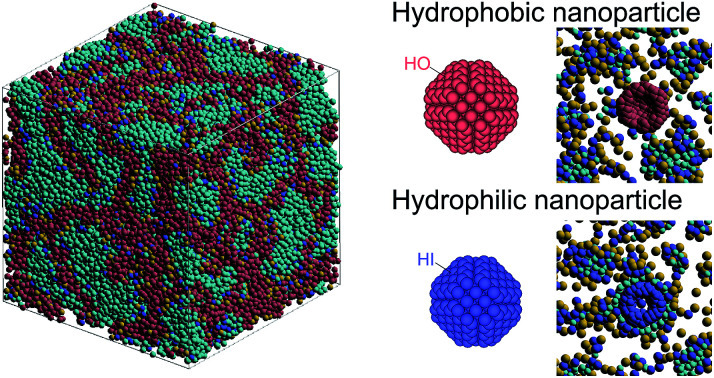

## 1 Introduction

In recent years, the world’s energy consumption has continued to increase, and we require clean energy sources with high efficiency. In particular, fuel cells provide high efficiency and discharge harmful gases such as carbon monoxide, nitrogen oxide and sulfur oxide in only extremely small amounts, unlike conventional fossil fuels. Hence, a solution to solve global environmental problems and energy resource issues, could be the development of fuel cells.^1–4^ In recent years, for example, Bae *et al.*^4^ demonstrated that an anode-supported fuel cell configuration based on yttrium-doped barium zirconate thin films could be used to develop highly efficient, physically and chemically stable PEFCs. This anode-supported fuel cell configuration allowed for a record high-power output of up to an order of magnitude higher than those of other reported fuel cells.

Polymer electrolyte fuel cells (PEFCs)^5–7^ have received considerable attention for their applications in fuel cell vehicles. The electrolytes of PEFCs have excellent physical properties, exhibiting stable temperatures and sufficiently low ion conductivity. It is also possible to miniaturize fuel cells and retain high power, relative to the fuel cell. Therefore, PEFCs are also expected to be applied in distributed power supply systems for domestic use.^8,9^ However, technological breakthroughs have not yet been discovered to accelerate the commercialization of PEFCs. It has been considered that the development of an epoch-making catalyst and the development of new characteristics of polymer electrolyte membranes are important issues in the practical use of PEFCs.^10,11^ Polymer electrolyte membranes that can easily satisfy the requirements of high proton conductivity, power generation durability, a low permeability of fuel, a capacity for resisting heat and other characteristics are required. Hence, the stabilized microphase-separated structure of polymer electrolyte membranes is a key aspect of PEFC performance.^12–15^ Jang *et al.*^12^ investigated the effect of the monomeric sequence (blocked or dispersed) of Nafion chains on the nanophase-segregation and transport in hydrated Nafion. They reported that the blocky sequence led to greater diffusion of water molecules than the dispersed sequence. Bae *et al.*^13^ synthesized a series of sulfonated poly(arylene ether sulfone ketone) multiblock copolymers with highly sulfonated hydrophilic blocks and measured the proton diffusion coefficient in the membranes. The membranes showed much higher proton conductivity than that of random and block copolymers over a wide range of humidities. Dorenbos and Morohoshi^14,15^ performed computer simulations of grafted and block polymer membranes with various hydrophobic and hydrophilic architectures. They found that pore morphologies strongly depended on the chain architecture. For the block polymers, longer hydrophilic blocks led to larger pores that were better connected, resulting in increased water diffusion.

In recent years, anisotropic nanoparticles which have complex shapes and chemical interactions were synthesized experimentally due to the progress in synthetic technology.^16,17^ Janus nanoparticles (JNP)^18–20^ have attracted a great deal of attention because they have two distinct surfaces with different properties. Recently, Pham^16^ reported that the morphology of the final particles could be controlled by the properties of cross-linked seeds on the initiators and monomers used in the polymerization. Therefore, we can expect that JNPs generate new specific properties and functions compared to homogeneous nanoparticles.^21,22^ Research on JNPs which affect membranes and vesicles as catalysts has proceeded by making full use of their characteristics.^23,24^ Many kinds of research have been conducted for realizing high-performance electrolyte membranes. However until now, a high-performance electrolyte membrane, with characteristics exceeding those of current fluorine-based membranes, has not yet been developed in the field of fuel cell vehicles. It is also known that the functionality of PEFC membranes is influenced by the self-assembled structure of the graft polymers.^25,26^ To our knowledge, a simulation study of the self-assembled structure of graft polymers using JNPs has not been reported in the literature. Therefore, we investigated the effect of hydrophilic (HI), hydrophobic (HO) and Janus nanoparticles on self-assembly of graft polymers by using molecular simulations.

## 2 Method

### 2.1 Dissipative particle dynamics (DPD) method

We adopt the dissipative particle dynamics (DPD) method^27–29^ to investigate self-assembly morphologies of PEFC, with and without various NPs, using in-house code. The DPD method has been proven to be an effective mesoscopic simulation tool to study fluid events occurring on millisecond timescales and micrometer length scales *via* tracking the motion of coarse-grained particles (composed of a group of atoms or molecules). Many researchers have studied the morphology of soft matter at mesoscopic level using the DPD method, for example, self-assembly of surfactant solutions,^30–32^ interactions between polymers and nanoparticles^24,33,34^ and phase separation for polymer solar cells.^35–37^

The fundamental equation in the DPD method is Newton’s equation of motion. Newton’s equation of motion for particle *i* is given by:1
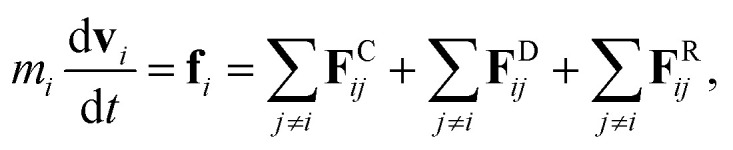
where *m* is the mass, **v** the velocity vector, **F**^C^ the conservative force, **F**^R^ the pairwise random force, and **F**^D^ the dissipative force. Note that each nanoparticle is treated as a rigid body,^38^ therefore all the DPD beads within the same nanoparticle have the same translational velocity, and the intra-JNP forces are not included in Newton’s equation of motion. The conservative force is softly repulsive and is given by2
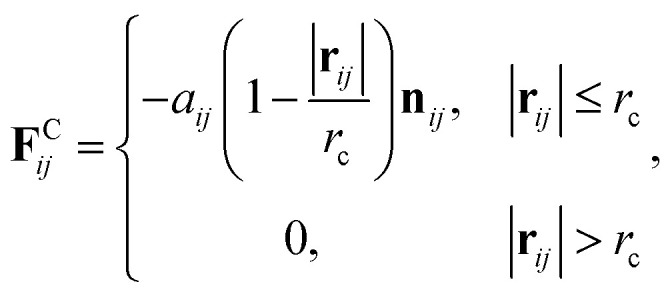
where **r** is the position vector, **r**_*ij*_ = **r**_*j*_ − **r**_*i*_, and **n**_*ij*_ = **r**_*ij*_/|**r**_*ij*_|. Here, *a*_*ij*_ is a parameter determining the magnitude of the repulsive force between particles *i* and *j*, and *r*_c_ is the cutoff distance. Random force (**F**^R^_*ij*_) and dissipative force (**F**^D^_*ij*_) are given by3
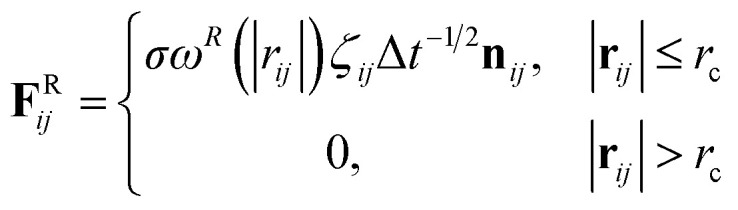
and4
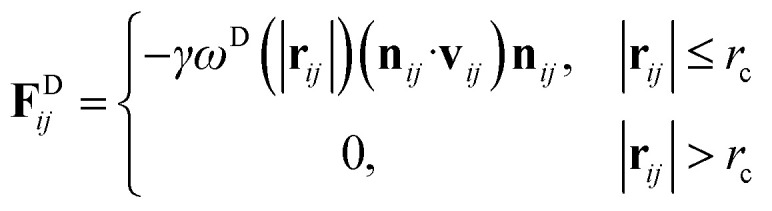
respectively, where **v**_*ij*_ = **v**_*j*_ − **v**_*i*_, *σ* is the noise parameter, *γ* is the friction parameter, and *ζ*_*ij*_ is a random number based on the Gaussian distribution. Here *ω*^R^ and *ω*^D^ are *r*-dependent weight functions which are given by5
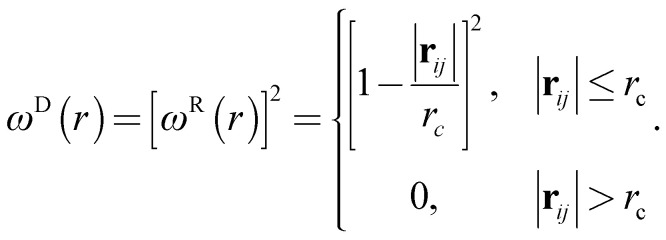


The temperature is controlled by a combination of dissipative and random forces. The noise parameter *σ* and friction parameter *γ* are connected to each other by the fluctuation-dissipation theorem in the following equation

6 *σ*^2^ = 2*γk*_B_*T*,

where *k*_B_ is the Boltzmann constant and *T* is the temperature. In the DPD simulation, the DPD interactions are soft and beads can easily overlap. Therefore, the polymer chain composed of DPD beads can have unphysical bond-crossing. A segmental repulsive potential (SRP) has been proposed to solve this issue by Pan and Manke.^39^ SRP can avoid unphysical bond-crossings by applying segmental repulsive forces between neighboring chains. However, the chain length of PEFC is set at 24 in our study and therefore the effect of entanglement is weak. Hence, we performed the DPD simulation to investigate self-assembled morphologies of PEFC without SRP.

### 2.2 PEFC, solvent, and NP models

**Fig. 1:**
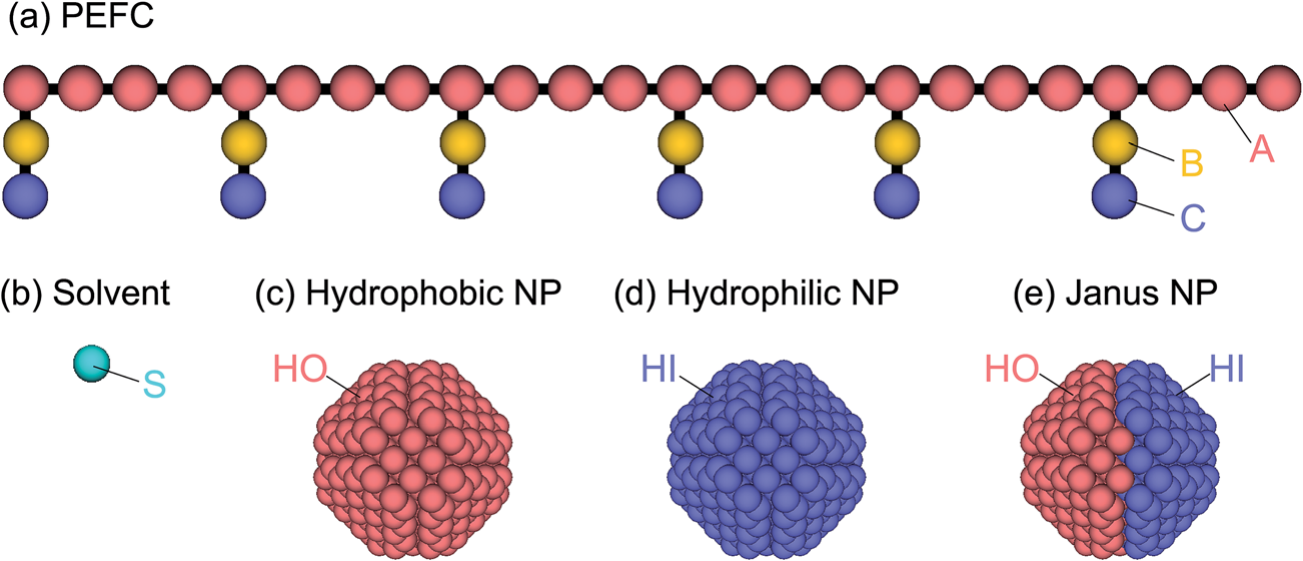
(a) The PEFC model is composed of a hydrophobic backbone (red) and amphiphilic side chains (yellow and blue). (b) The solvent (water) model is a single DPD bead (aqua). (c, d, e) Three kinds of NP models are employed in this simulation: the hydrophobic uniform NP (c), the hydrophilic uniform NP (d), and the Janus NP (e).

The PEFC model contains a hydrophobic backbone and amphiphilic side chains that are connected with harmonic springs as shown in Fig. 1(a). The backbone (red), hydrophobic groups in the side chain (yellow), and hydrophilic groups in the side chain (blue) are labeled by the letters A, B, and C, respectively. The spring force (**F**^S^_*ij*_) is given by

7 **F**^S^_*ij*_ = −*k*(|**r**_*ij*_| − *r*_s_)**n**_*ij*_

where *k* is the spring constant and *r*_s_ is the equilibrium bond distance between the *i* and *j* beads. The particle density *ρr*_c_^3^ is 3. A solvent (water) molecule is represented by a single bead labeled by the letter S.

To study the effect of a change in the chemical surface of the NPs, we considered three NPs so that we could change the hydrophobic–hydrophilic balance of the NP (Fig. 1c–e). Hereafter, the hydrophobic and hydrophilic beads in NPs are labeled HO and HI, respectively. Moreover, to investigate the effect of the nanoparticles’ density on the self-assembled structures, we introduced three different amounts of NPs (*N*_NP_): 1, 4, and 8. The initial configuration of NPs for the simulation was random. The NPs consisted of hydrophobic and/or hydrophilic DPD beads on a diamond lattice with a lattice constant, *α* = 0.73. Each NP consisted of 538 DPD beads, and the radius was 2.0. For the JNP, 257 beads were hydrophobic and another 281 were hydrophilic. Note that the numbers are not equal because the diamond lattice cannot be halved (in the same plane and with the same number of beads).

The repulsions between any two beads in the solution are shown in Table 1. The interaction parameters are adopted from an earlier study which investigated the structure of hydrated Nafion membranes using the DPD method.^40^ Here, it is known that the interaction parameters for the conservative force between any two beads are related to the Flory–Huggins *χ*-parameters. The Flory–Huggins *χ*-parameters between DPD particles were estimated from the mixing energy calculation using an atomistic simulation. The interaction parameters for different kinds of beads (*a*_*ij*_) were obtained by the equation *a*_*ij*_ = *a*_*ii*_ + 3.268*χ*_*ij*_ where *χ* is the Flory–Huggins parameter when the number density *ρ* = 3.0. To ensure that these *χ*-parameters were physically sensible, like Yamamoto and Hyodo^40^ we calculated partial atomic charges of the PEFC monomer and water, with the assumption that the system involved electrostatic interactions. In our simulation, the total numbers of beads were varied from 81 000 to 85 304, depending on *N*_NP_. Three PEFC concentrations were examined, 70%, 80%, and 90%; and the total number of polymers within the simulation box for each PEFC concentration was 1575, 1800, and 2025 respectively. The remaining beads were solvent. The dimensions of the simulation box were 30 × 30 × 30. The *r*_s_ was set at 0.86 and the spring constant *k* at 100*k*_B_*T*/*r*_c_^2^.^40^ The noise parameter *σ* was set to 3.0, the friction parameter *γ* was set to 4.5, and the time step d*t* was 0.04. The periodic boundary condition was applied in all three dimensions. All simulations were performed in a constant volume and constant temperature ensemble. The temperature was set to 1.0*k*_B_*T*. The DPD unit of length was the cutoff radius, *r*_c_, the unit of mass was the bead mass, *m*, and the unit of energy was *k*_B_*T*.

**Table 1:** Interaction parameters *a*_*ij*_ (in *k*_B_*T*/*r*_c_ units) between bead pairs in eqn (2)

	A	B	C	S	HO	HI
A	104.0	104.1	114.2	122.9	104.0	150.0
B	104.1	104.0	108.5	120.0	104.0	150.0
C	114.2	108.5	104.0	94.0	150.0	104.0
S	122.9	120.0	94.0	104.0	150.0	104.0
HO	104.0	104.0	150.0	150.0	104.0	150.0
HI	150.0	150.0	104.0	104.0	150.0	104.0

## 3 Results and discussion

**Fig. 2:**
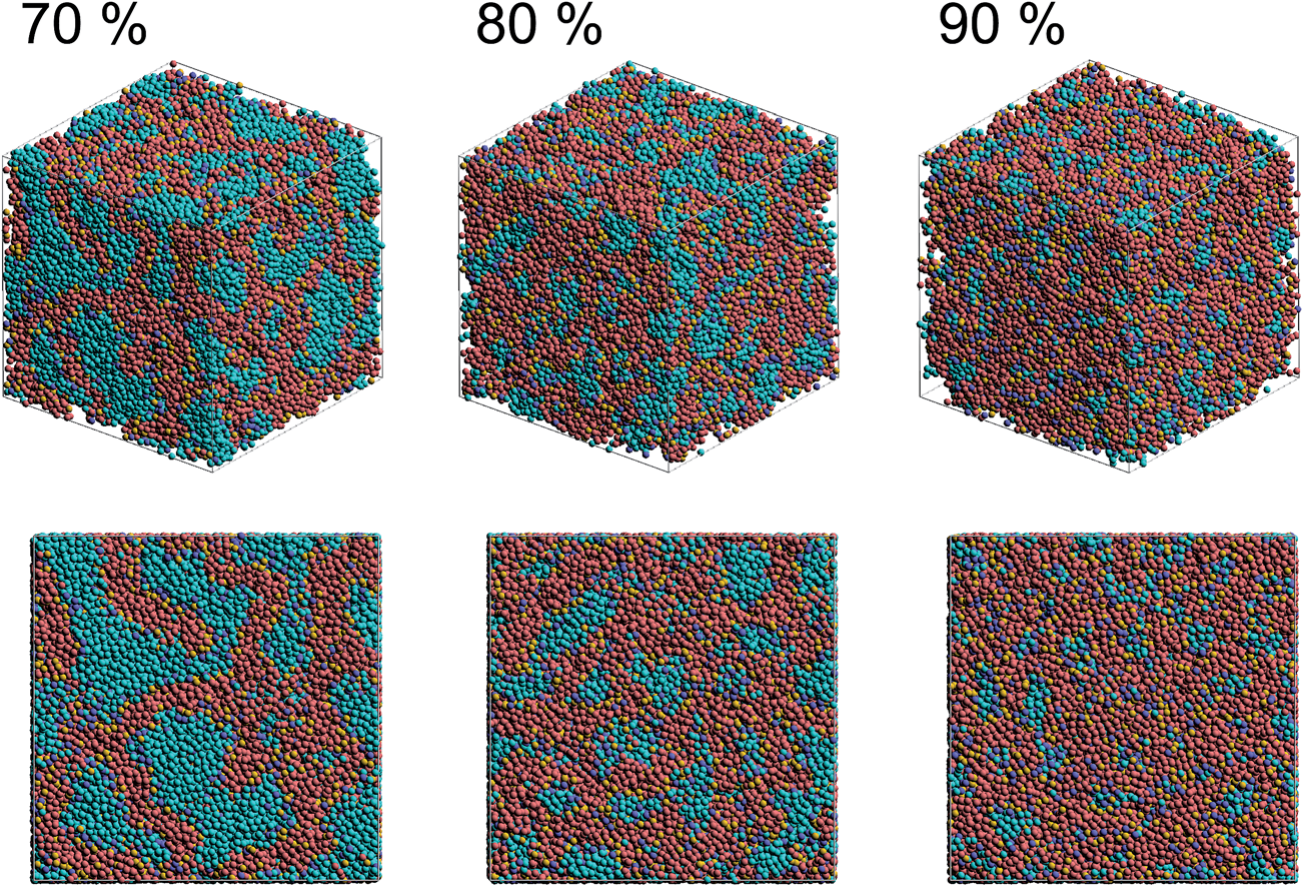
Snapshots of equilibrium morphologies of PEFC membrane systems without NPs for polymer concentrations of 70, 80, and 90% at *t* = 4.0 × 10^4^.

**Fig. 3:**
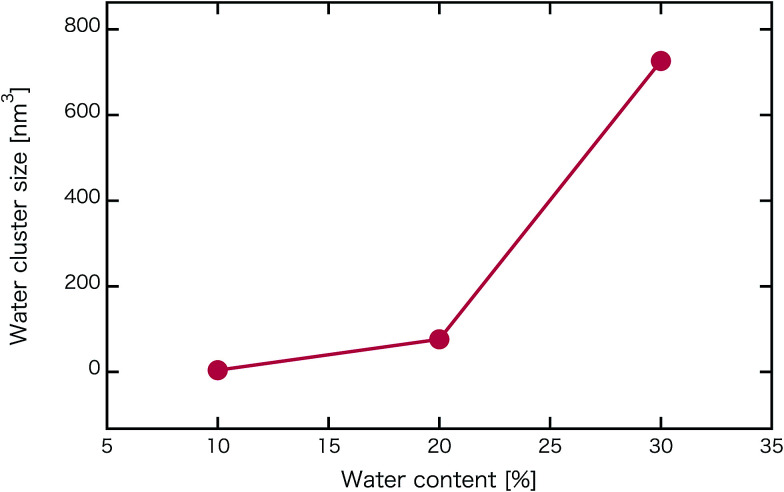
Water content dependence of the mean water cluster size of PEFC membrane systems without NPs.

Fig. 2 shows self-assembled morphologies of PEFC without (W/O) NPs. The water cluster size for equilibrium structures at each concentration of PEFC is shown in Fig. 3. Here, the water cluster size was estimated from the number of DPD particles forming water clusters. Note that we set three water molecules as coarse-grained for a single DPD particle, and accordingly, the unit of mass was 54 in atomic units. The three DPD particles were contained in a cube of *r*_c_^3^ and therefore corresponded to a volume of 270 Å^3^ due to the volume of a water molecule being 30 Å^3^.^41^

**Fig. 4:**
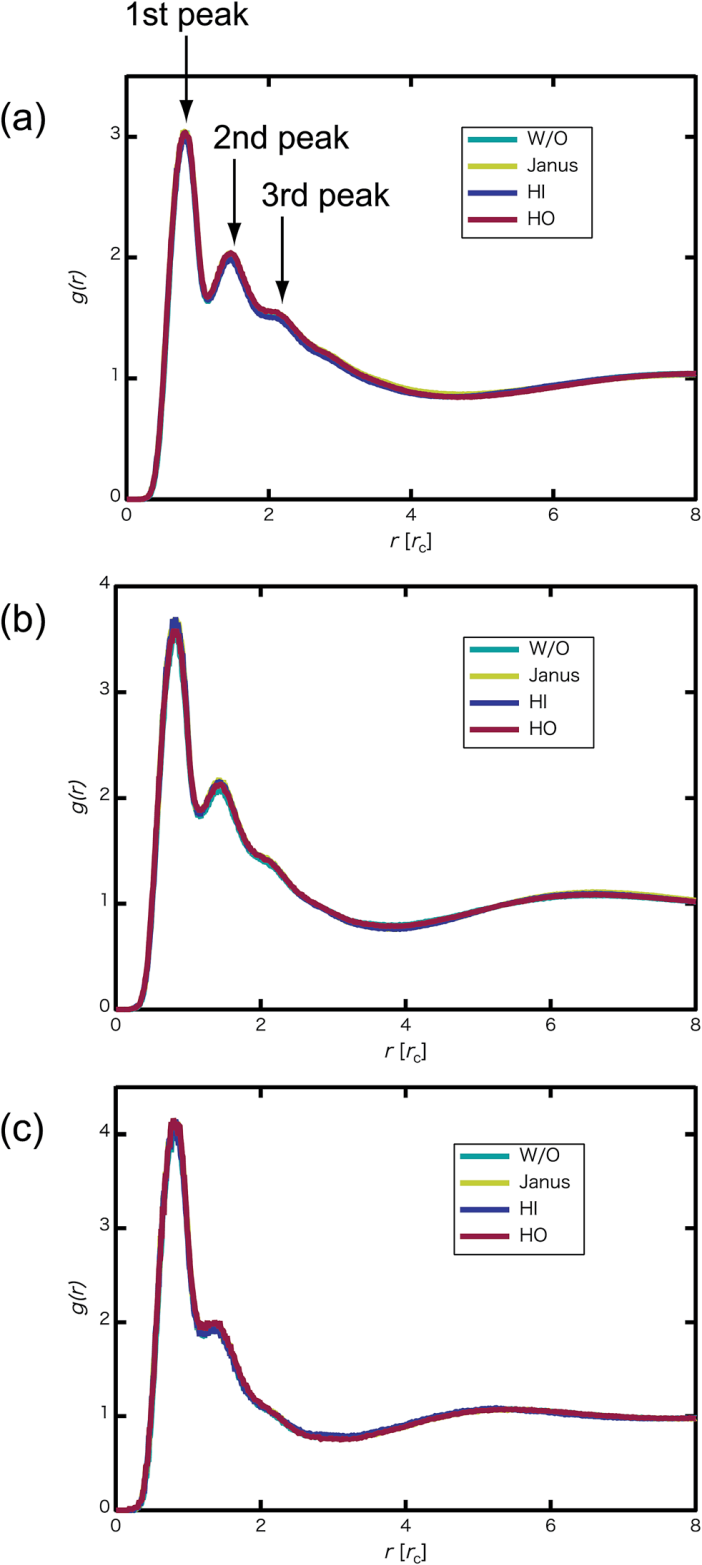
(a) RDF of water particles *g*(*r*) for the 70% PEFC membrane system at *t* = 4.0 × 10^4^. (b) RDF of water particles for the 80% PEFC membrane system at *t* = 4.0 × 10^4^. (c) RDF of water particles for the 90% PEFC membrane system at *t* = 4.0 × 10^4^. Aqua: Without NPs, yellow: Janus nanoparticles, blue: Hydrophilic nanoparticles, and red: Hydrophobic nanoparticles. The number of NPs in each system was 8.

**Fig. 5:**
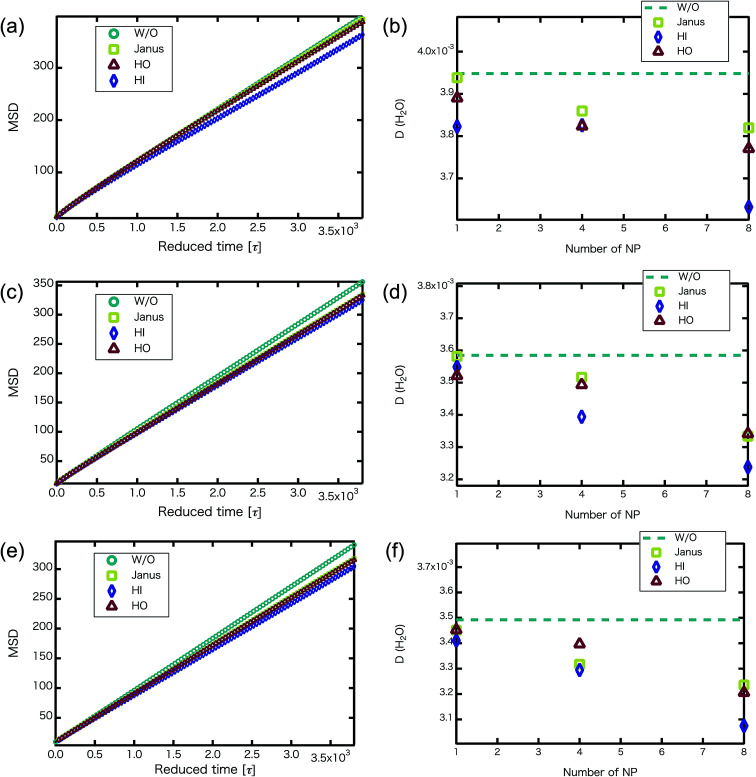
(a, c and e) For the *N*_NP_ = 8, MSD *versus* reduced simulation time of water particles (without NPs : circles, with Janus NPs : squares, with HO NPs : triangles, with HI NPs : diamonds) after equilibrium. (b, d and f) D(H_2_O) (without NPs : dotted line, with Janus NPs : squares, with HI NPs : diamonds, with HO NPs : triangles) plotted against the number of nanoparticles. (a and b) MSD of water particles and D(H_2_O) for the 70% PEFC membrane system. (c and d) MSD of water particles and D(H_2_O) for the 80% PEFC membrane system. (e and f) MSD of water particles and D(H_2_O) for the 90% PEFC membrane system. A 95% confidence interval was estimated from the result of independent simulations. We confirmed that the fluctuation of the D(H_2_O) value was extremely small.

We confirmed that as the water content increased, water cluster size was also clearly increased. The behavior of the water cluster size was in reasonable agreement with an earlier study.^40^ We calculated the radial distribution function (RDF) of water particles (see Fig. 4). In Fig. 4(a) and (b), three peaks arise in the range of *r* < 2.0. On the other hand, the third peak is not observed in Fig. 4(c). As a result, differences appear among the self-assembled structures of water depending on the concentration of PEFC. Here, in order to identify the effect of nanoparticles (NPs) for self-assembled structures, we calculated the time-averaged cluster size of water 〈*N*_W_〉 for all systems. When the PEFC concentration was 70%, 〈*N*_W_〉 decreased by adding NP. For the PEFC concentrations of 80% and 90%, the tendency for decreasing 〈*N*_W_〉 values was not observed. This is because 〈*N*_W_〉 for 80% and 90% was already very small before adding NPs. Therefore, significant differences in 〈*N*_W_〉 did not appear by adding NPs. According to Fig. 4, similar peaks formed regardless of whether we added NPs to the PEFC membrane or not. Hence, we considered that the effect of NPs on the internal structure of the water clusters was weak. We calculated the time-averaged mean square displacement (MSD) of water for the NPs of different chemical nature (Fig. 5(a) (PEFC concentration of 70%), (c) (PEFC concentration of 80%) and (e) (PEFC concentration of 90%)). Although for DPD, with its simple soft potentials, it is a real challenge to correctly describe the diffusion of molecules through condensed phases, we believe that it is possible to qualitatively confirm the diffusion data.^42^ The MSD is given by8
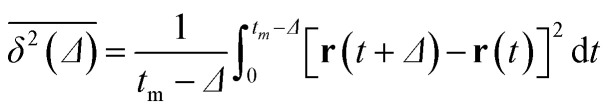
where *Δ* is the lag time, *t*_m_ is the total measurement time and **r** is a position of the particle at time *t*. Moreover, the diffusion constant of water (D(H_2_O)) was obtained from the slopes of the MSD curves (Fig. 5(b) (PEFC concentration of 70%), (d) (PEFC concentration of 80%) and (f) (PEFC concentration of 90%)). As is evident from Fig. 5, when the *N*_NPs_ is more than 4 in the PEFC membrane, D(H_2_O) with various NPs is lower than D(H_2_O) without various NPs. It seems that the D(H_2_O) behavior is closely related to the position of the NPs in the PEFC membrane–water system. In the case of adding HO NPs, HO NPs were incorporated into the PEFC membrane because HO NPs do not prefer to be in contact with water particles. In the case of adding JNPs, the hydrophilic surfaces of the JNPs prefer to be in contact with the water particles and the hydrophobic surfaces of the JNPs prefer to be in contact with the hydrophobic beads of PEFC. Moreover, in the case of adding HI NPs, HI NPs were incorporated into the water-phase because HI NPs prefer to be in contact with the water particles. Interestingly, despite the respective NPs (Janus, HO, and HI) being located in different positions in the PEFC membrane system, we saw that there was a declining trend in D(H_2_O). HI NPs had the biggest effect of suppressing the diffusion of water because HI NPs were incorporated into the water-phase. It was also seen that D(H_2_O), with various NPs, gradually decreases as the *N*_NPs_ increases for the three PEFC concentrations (70%, 80%, and 90%). Since it has been reported that the diffusion coefficient of protons has a strong mutual relationship with the diffusion coefficient of water,^43^ an improvement of water diffusion corresponds to that of proton conductivity. This means that a decrease in proton conductivity is obtained by adding various NPs.

We confirmed that the MSD of water behavior that was obtained was similar to that in earlier studies. Jang *et al.*^12^ reported that the monomeric sequence of Nafion chains affects the transport in hydrated Nafion. They used two extreme monomeric sequences, one of which was blocky and the other dispersed, to investigate such properties. In the case of the blocky Nafion, the MSD of water was higher than for the dispersed sequence. Thus, we found that adding various NPs is equivalent to using the blocky Nafion for influencing the MSD of water behavior. Moreover, it is reported that the D(H_2_O) of the grafted polymer is higher than that of the block polymer.^14^ In other words, as the ratio of the length of the side chains and the inter-branching distance gradually decreases, the D(H_2_O) increases. In our simulation, similar behavior of D(H_2_O) is observed when we added various NPs into the PEFC membrane.

**Fig. 6:**
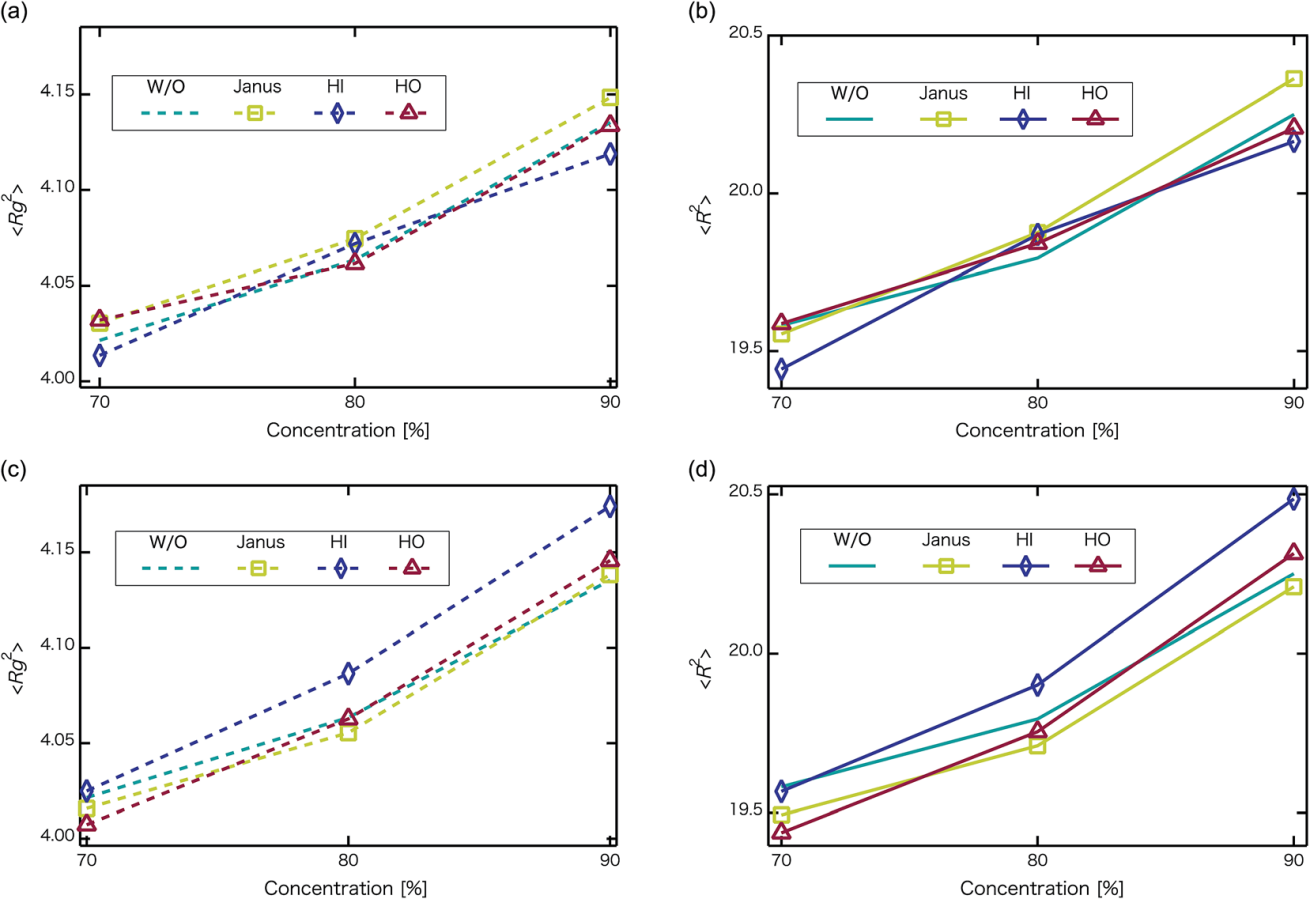
Concentration dependence of mean square radius of gyration 〈*R*_g_〉 and mean square end-to-end distance 〈*R*〉 at *t* = 4.4 × 10^4^. The upper left panel is 〈*R*_g_^2^〉 for when the number of NPs in the system is 4 (without NPs :dotted line, with Janus NPs : squares, with HI NPs : diamonds, with HO NPs : triangles). The upper right panel is 〈*R*^2^〉 for when the number of NPs in the system is 4 (without NPs : line, with Janus NPs : squares, with HI NPs : diamonds, with HO NPs : triangles). The lower left panel is 〈*R*_g_^2^〉for when the number of NPs in the system is 8 (without NPs : dotted line, with Janus NPs: squares, with HI NPs: diamonds, with HO NPs: triangles). The lower right panel is 〈*R*^2^〉 for when the number of NPs in the system is 8 (without NPs : line, with Janus NPs : squares, with HI NPs : diamonds, with HO NPs : triangles). We performed 3 independent simulations under each condition to display typical measurement uncertainties of the data. A 95% confidence interval was estimated from the result of the independent simulations. We confirmed that the fluctuation of the 〈*R*_g_〉 and 〈*R*^2^〉 were extremely small.

**Fig. 7:**
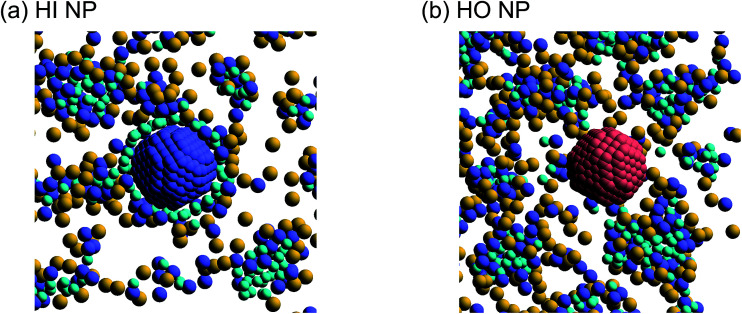
(a) A snapshot of self-assembled PEFC structures located near the HI NPs. (b) A snapshot of self-assembled PEFC structures located near the HO NPs. The hydrophobic backbone (red) molecules are removed for clarity.

Next, self-assembled structures of PEFC were considered. Fig. 6 shows the mean square radius of gyration 〈*R*_g_〉 and the mean square end-to-end distance 〈*R*〉. In our simulation, we used the end-to-end distance of the hydrophobic backbone to calculate 〈*R*〉. For *N*_NP_ = 0, 4, and 8, 〈*R*_g_〉 and 〈*R*〉 increase with PEFC concentration, independent of the NP property. Here, we observed a clear difference in the value of 〈*R*_g_〉 and 〈*R*〉 in the case of adding HI NPs. For the system where *N*_NP_ = 8 and HI NPs were added with both PEFC concentrations of 80%, and 90%, the 〈*R*_g_〉 and 〈*R*〉 showed maximum values compared to all other systems. The reason for this difference can be explained as follows. It seems that only for *N*_NP_ = 8, HI NPs can contact the hydrophilic beads (Fig. 7(a)). In other words, there is too little water available to incorporate the HI NPs into water-phase. Therefore, in the case of adding HI NPs, we observed a clear difference in the value of the 〈*R*_g_〉 and 〈*R*〉 in comparison to others as PEFC concentration increases. In contrast, in the case of adding JNPs, the 〈*R*_g_〉 and 〈*R*〉 have different values from those values obtained when adding HI NPs. The hydrophilic superficial area of the JNP is smaller than that of the HI NPs. In comparison with the case of when we added HI NPs, the extent to which JNPs contact the hydrophilic side chain groups decreases. Moreover, HO NPs cannot contact the hydrophilic beads (Fig. 7(b)) Hence, in both cases of adding either JNPs or HO NPs, lower values of 〈*R*_g_〉 and 〈*R*〉 are observed in comparison to the case where HI NPs are added. For the systems where *N*_NPs_ = 4, the reason why 〈*R*_g_〉 and 〈*R*〉 are not of the highest values when HI NPs are added, can be explained as follows. The HI NPs have a greater level of incorporation into the water particles system.

## 4 Conclusions

We have applied DPD to investigate the effect of hydrophilic, hydrophobic and Janus nanoparticles on the self-assembly of graft polymers. As the concentration of PEFC was increased, water cluster size was also clearly increased. The RDF of water particles showed that differences appear among the self-assembled structures of water depending on the concentration of PEFC. Moreover, we calculated the time-averaged cluster size of water (〈*N*_W_〉) to identify the effect of nanoparticles on self-assembled structures. Only a PEFC concentration of 70%, gave a decrease in 〈*N*_W_〉 by adding nanoparticles. In contrast, we did not observe differences of 〈*N*_W_〉 for PEFC concentration of 80% and 90%. Here, Fig. 4 showed that similar peaks formed regardless of whether we add nanoparticles to the PEFC membrane or not. Thus, the effect of nanoparticles on the internal structure of water clusters was weak. On the other hand, the MSD of water and D(H_2_O) showed a different tendency. There was a close relationship between the position of the NPs within the PEFC membrane and its chemical design. We also focused on self-assembled PEFC structures. A clear difference in the value of 〈*R*_g_〉 and 〈*R*〉 was observed. In particular, when the *N*_NPs_ = 8, the HI NPs had less space within which to incorporate into the water particles. As a result, in the case of adding HI NPs, the 〈*R*_g_〉 and 〈*R*〉 showed the highest values compared to all others. As *N*_NPs_ increases, it is suggested that the effect of the change in the chemical surface of the nanoparticles gradually becomes stronger.

## Conflicts of interest

There are no conflicts to declare.

## Supplementary Material
